# Risk factors for type 2 diabetes mellitus in Chinese rheumatoid arthritis patients from 2018 to 2022: a real-world, single-center, retrospective study

**DOI:** 10.3389/fimmu.2024.1445639

**Published:** 2024-10-04

**Authors:** Ruomeng Pei, Jia Wang, Peifeng He, Qi Yu, Shengxiao Zhang, Gaoxiang Shi, Geliang Liu, Xiaofeng Li

**Affiliations:** ^1^ Third Hospital of Shanxi Medical University, Shanxi Bethune Hospital, Shanxi Academy of Medical Sciences, Tongji Shanxi Hospital, Taiyuan, China; ^2^ School of Management, Shanxi Medical University, Taiyuan, China; ^3^ Institute of Medical Data Science, Shanxi Medical University, Taiyuan, China; ^4^ Shanxi Key Laboratory of Big Data for Clinical Decision, Shanxi Medical University, Taiyuan, China; ^5^ Department of Rheumatology, The Second Hospital of Shanxi Medical University, Taiyuan, Shanxi, China; ^6^ Key Laboratory of Cellular Physiology, Ministry of Education, Shanxi Medical University, Taiyuan, China; ^7^ School of Basic Medical Sciences, Shanxi Medical University, Taiyuan, China

**Keywords:** rheumatoid arthritis, type 2 diabetes mellitus, helper T cells, regulatory T cells, risk factors

## Abstract

**Introduction:**

In patients with rheumatoid arthritis (RA), the increased risk of concomitant type 2 diabetes mellitus (T2D) is an important contributor to increased mortality and decreased quality of life; however, the mechanisms and pathogenetic factors remain unknown.

**Methods:**

In this study, we aimed to assess the risk factors for T2D in patients with RA. We recruited 206 healthy controls and 488 patients with RA, 160 of whom had comorbid T2D. General clinical information, disease characteristics, and circulating lymphocyte levels detected using modified flow cytometry were collected from all participants. Logistic regression models adjusted for confounders were fitted to estimate the risk factors of T2D in patients with RA.

**Results:**

The incidence of RA in patients with T2D was 15.6%. Patients with RA and T2D had a longer disease duration, higher BMI, and a higher incidence of hypertension and a family history of diabetes than those with RA but no T2D. The absolute numbers of T helper 2 cell (Th2) and Regulatory T cells (Treg) decreased in patients with RA and T2D, which led to an increase in the ratios of Th1/Th2 and Th17/Treg cells. Multivariate logistic regression analysis showed that a family history of diabetes, a higher incidence of hypertension, higher neutrophil-lymphocyte ratio (NLR) levels, lower platelet-lymphocyte ratio (PLR) levels, and fewer circulating Th2 and Treg cells were associated with an increased risk of T2D in patients with RA.

**Discussion:**

The levels of peripheral lymphocytes, especially Th2 and Treg cells, are closely related to the occurrence of T2D in patients with RA; however, the influence of body mass index (BMI), family history of diabetes, and systemic inflammation should not be ignored.

## 1 Introduction

Rheumatoid arthritis (RA) is an autoimmune disease characterized by persistent synovial inflammation and bone damage; the diseases affects 1% of the global population (1). While several factors such as genetics, environment, and immunity, are undoubtedly involved in the occurrence and progression of RA, evidence for its pathogenesis confirm the crucial role of immune cells (2). Although the long-term prognosis of RA has improved to some extent owing to the emergence of multiple treatment modalities, the life expectancy of patients with RA is still significantly lower than that of the general population because of the emergence of extra-articular manifestations and various comorbidities, especially type 2 diabetes mellitus (T2D) (3, 4). Diabetes mellitus (DM) is a chronic systemic disease characterized by elevated blood sugar levels and ocular, renal, and vascular involvement, which can result in systemic inflammation, impaired glucose metabolism, and potentially life-threatening complications (5).

Although the relevant mechanisms remain elusive, increasing evidence indicates that patients with RA are more likely to develop DM, with a prevalence rate of approximately 6%-14%; this not only increases the long-term mortality of patients with RA but also aggravates the original metabolic disorders of DM (6–9). The abnormal immune response mediated by unbalanced lymphocyte subgroups such as CD4^+^ T subpopulations during the occurrence and development of RA not only damages bones and joints but also affects metabolic organs, including the liver. This results in glucose metabolism disorders and participating in the development of DM (10). However, thus far, no reports have detailed the changes in circulating lymphocyte population levels in patients with RA and DM.

In this study, we collected data on patient demographics, disease characteristic, and the absolute numbers and proportions of peripheral lymphocytes, especially CD4^+^ T subsets, of patients with RA and DM and those with RA but without DM (RA-T2D and RA-N-T2D, respectively), and analyzed the factors influencing the occurrence of DM in patients with RA. We hope our findings provide new ideas for early clinical prevention and treatment.

## 2 Methods

### 2.1 Study population

**Figure 1:**
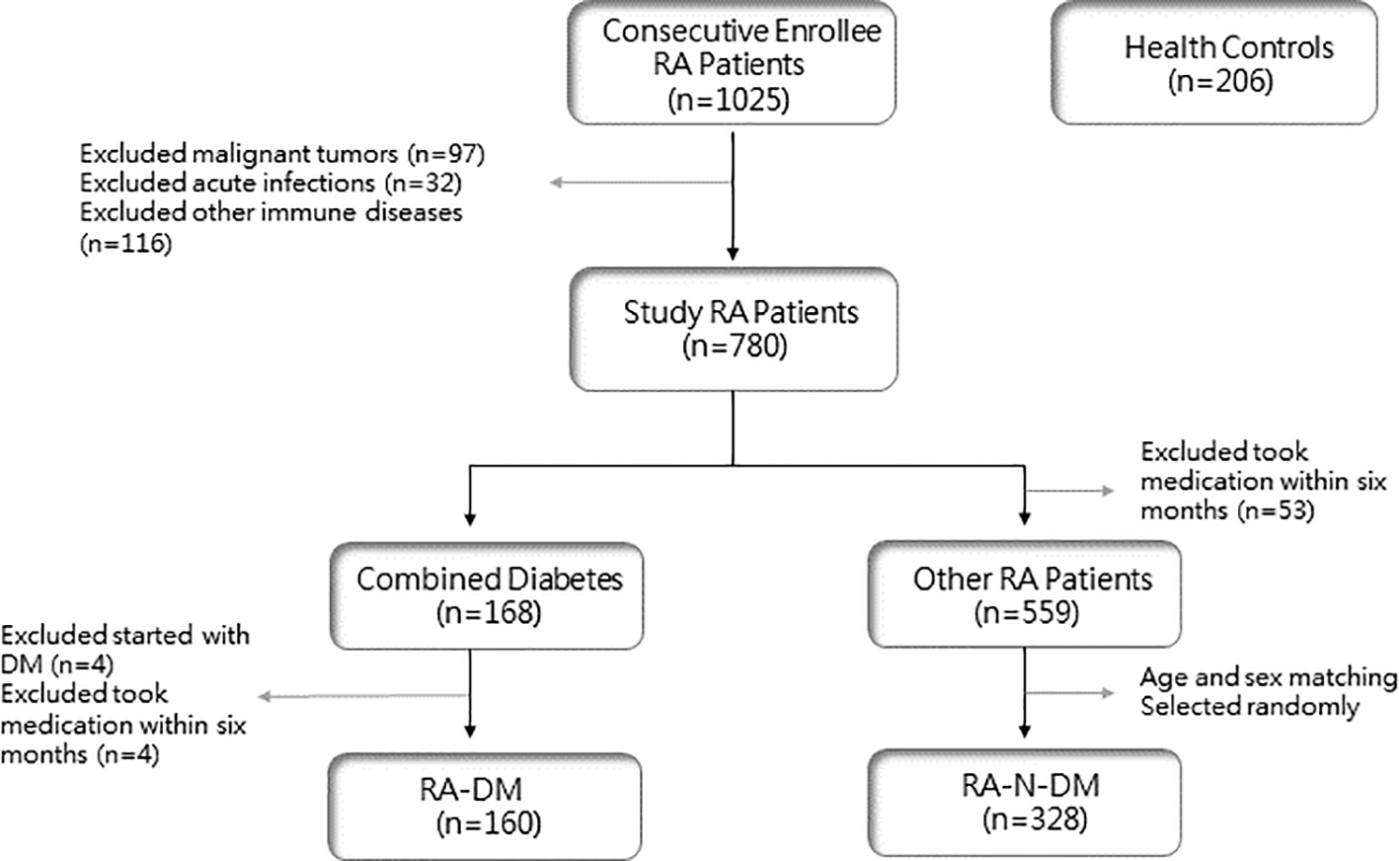
Flow chart of patient enrolment. RA, rheumatoid arthritis; DM, diabetes mellitus.

The study population was selected from 1025 patients with RA who were consecutively enrolled between January 2018 and June 2022, all of whom met the 1987 ARA criteria and the 2010 EULAR revised RA classification diagnostic criteria (11, 12). Patients with RA with other rheumatic diseases, malignant tumors, and acute and chronic infections were excluded, and 780 patients who met the inclusion criteria were enrolled. Among them, 719 patients did not take hormones, DMARDs, or other drugs that may have affected the number of peripheral blood lymphocytes for at least six months. Based on the diagnostic criteria for T2D published by the World Health Organization (WHO) in 1999 (13), 160 patients were included in the RA-DM group (108 females, 52 males). Among the remaining 559 patients with RA, 328 age- and sex-matched patients were randomly selected as the RA-N-DM group (223 females and 105 males). In addition, 206 healthy controls (136 females and 70 males) were enrolled from the rheumatology clinic or physical examination center of the Second Hospital of Shanxi Medical University (**Figure 1**).

### 2.2 Data collection

General clinical information of patients with RA and healthy controls was collected, including sex, age, disease duration, height, weight, calculated BMI, smoking status, alcohol consumption, hypertension, osteoporosis, history of nephropathy, and family history of diabetes. Indicators of disease activity, such as tender joint count (TJC), swollen joint count (SJC), and disease activity score in 28 joints (DAS28), were recorded for all patients. The general laboratory indicators included in the analysis were erythrocyte sedimentation rate (ESR), C-reactive protein (CPR), immunoglobulin G (IgG), immunoglobulin A (IgA), immunoglobulin M (IgM), white blood cell (WBC), red blood cell (RBC), platelet (PLT), lymphatic (LYM), monocytes (MO), neutrophilic granulocyte (NE), platelet-lymphocyte ratio (PLR), monocytes- lymphatic ratio (LMR), neutrophil-lymphocyte ratio (NLR), systemic immune-inflammation index (SII), blood urea nitrogen (BUN), serum creatinine (Cr), fasting blood glucose (FBG), albumin (ALB), globulin (GLB) and albumin/globulin ratio (A/G). Simultaneously, the absolute counts and proportions of T, B, CD8^+^ T, natural killer (NK), and CD4^+^ T cells and their subsets, such as T helper 1 (Th1), Th2, Th17, and regulatory T (Treg) cells, were recorded in detail for all recruiters.

### 2.3 Statistical analysis

IBM SPSS Statistics version 22 was used for all statistical analyses. The sample size of each group was analyzed by power analysis to ensure its validity. Percentages and mean values with standard deviations (SD) were used to describe the demographic information of all the subjects. Medians and quartiles were used to describe the disease characteristics of all patients. The chi-square test was used for categorical variables, whereas the t-test, Mann–Whitney U test, and Wilcoxon test were used for continuous variables. Factors associated with T2D in patients with RA were assessed using univariate and multivariate logistic regression analyses. *p*<0.05 indicated that the difference was statistically significant.

## 3 Results

### 3.1 Demographic features and general information

There was no significant difference in sex (χ^2 ^= 0.227, *p*>0.05) and mean age (t = 3.242, *p*>0.05) between patients with RA (including 328 RA-N-DM patients and 160 RA-DM patients) and 206 healthy controls. Compared with RA-N-DM group, baseline demographics information including disease duration (Z=*−*4.571, *p<*0.001), body weight (Z=*−*7.736, *p<*0.001), BMI (Z=*−*6.8, *p<*0.001), the proportion of combined hypertensive (Z=9.434, *p<*0.01), history of nephropathy (Z=-2.078, *p*<0.05) and family history of diabetes (Z=5.541, *p<*0.001) in RA-DM group were increased (**Table 1**). This suggests that the combination of T2D may affect the vascular function, renal function, and nutritional status of patients with RA and increase their medical costs.

**Table 1:** A summary of baseline demographics of all enrolled patients.

	RA-N-DM	RA-DM	X^2^/t/Z	*p*-value
N	328	160		
Sex (female/male)	223/105	108/52	0.227	0.893
Age (years), mean (SD)	61.02 ± 9.35	62.71 ± 9.24	3.242	0.072
Disease duration (years), mean (SD)	7.67 ± 7.67	11.64 ± 10.32	-4.571	<**0.001**
Height (cm), mean (SD)	161.2 ± 7.31	159.39 ± 7.25	-1.944	0.052
Weight (kg), mean (SD)	60.28 ± 9.82	65.07 ± 8.4	-7.736	<**0.001**
BMI, mean (SD)	21.76 ± 5.41	24.52 ± 6.74	-6.8	<**0.001**
DAS28	4.4 ± 0.5	4.5 ± 0.5	-1.928	0.054
Smoking, n (%)	51 (15.5)	33 (20.6%)	1.945	0.163
Alcohol consumption, n (%)	16 (4.9)	14 (8.8%)	2.794	0.095
Hypertension, n (%)	103 (31.4)	73 (45.6%)	9.434	**0.002**
Osteoporosis, n (%)	70 (21.3)	41 (25.6%)	1.123	0.289
Nephropathy, n (%)	19 (5.8)	19 (11.9)	5.541	<**0.001**
Family history of diabetes, n (%)	27 (8.2)	37 (23.1)	20.934	<**0.001**

Bold values in the summary indicate significant p values <0.05.

N, number of patients; SD, standard deviation; BMI, Body mass index; DAS28, disease activity score 28S.

### 3.2 Disease characteristics

Compared with RA-N-DM group, the levels of IgG (Z=*−*2.826, *p<*0.05), IgM (Z=*−*2.827, *p<*0.01), ALB (Z=*−*4.957, *p<*0.001) and A/G (Z=*−*3.095, *p<*0.05) in RA-DM group were significantly reduced, CRP (Z=*−*2.078, *p<*0.05), FBG (Z=*−*10.85, *p<*0.001) and GLB (Z=*−*2.349, *p<*0.05) were increased. There was no significant difference in ESR (Z=*−*1.412, *p>*0.05), IgA (Z=*−*0.091, *p>*0.05), WBC (Z=*−*1.39, *p>*0.05), RBC (Z=*−*1.123, *p>*0.05), PLT (Z=*−*1.942, *p>*0.05), LYM (Z=*−*1.38, *p>*0.05), MO (Z=*−*0.097, *p>*0.05), NE (Z=*−*1.861, *p>*0.05), PLR (Z=*−*0.372, *p>*0.05), LMR (Z=*−*1.496, *p>*0.05), NLR (Z=*−*1.928, *p>*0.05), SII (Z=*−*0.479, *p>*0.05), BUN (Z=*−*1.728, *p>*0.05) and Cr (Z=*−*1.278, *p>*0.05) between group RA-N-DM and RA-DM (**Table 2**). This suggests that patients with RA and T2D may have more severe systemic inflammatory reactions and immunoregulatory function disorders.

**Table 2:** A summary of disease characteristics of patients with RA, median (Q1, Q3).

	RA-N-DM	RA-DM	Z	*p*-value
ESR (mm/h)	46 (27, 83)	53.5 (27.75, 94)	*−*1.412	0.158
CRP (mg/L)	15.9 (5.63, 43.6)	18.9 (6.55, 68.1)	*−*2.078	**0.038**
IgG (g/L)	13 (10.4, 16.2)	11.8 (8.27, 14.95)	*−*2.826	**0.005**
IgA (g/L)	2.95 (2.06, 3.99)	2.86 (2.01, 4.09)	*−*0.091	0.927
IgM (g/L)	1.29 (0.91, 1.76)	1.06 (0.79, 1.59)	*−*2.827	**0.005**
WBC (×10^9^/L)	6.74 (5.38, 8.35)	7.37 (5.41, 8)	*−*1.39	0.165
RBC (×10^12^/L)	4.18 (3.9, 4.49)	4.17 (3.7, 4.57)	*−*1.123	0.261
PLT (×10^9^/L)	279 (221, 358.75)	263 (198.25, 340.75)	*−*1.942	0.052
LYM (×10^9^/L)	1.63 (1.33, 2.18)	1.55 (1.15, 2.22)	*−*1.38	0.167
MO (×10^9^/L)	0.5 (0.33, 0.64)	0.48 (0.35, 0.63)	*−*0.097	0.923
NE (×10^9^/L)	4.27 (3.27, 5.64)	4.88 (3.41, 6.19)	*−*1.861	0.063
PLR	159.64 (118.6, 228.95)	160.89 (109.24,235.04)	*−*0.372	0.71
LMR	3.56 (2.59, 5.06)	3.41 (2.46, 4.73)	*−*1.496	0.135
NLR	2.5 (1.85, 3.63)	2.8 (2.03, 4.32)	*−*1.928	0.054
SII	716.39 (461.65, 1125.16)	738.3 (464.47, 1291.05)	*−*0.479	0.632
BUN (mg/dL)	5.15 (4.2, 6.2)	5.5 (4.13, 7.1)	*−*1.728	0.084
Cr (mg/dL)	54.2 (47.25, 62)	53 (44, 63.75)	*−*1.278	0.201
FBG (mmol/L)	4.83 (4.33, 5.38)	6.71 (5.34, 8.82)	*−*10.85	<**0.001**
ALB (mg/L)	37.15 (33.9, 40.23)	34.25 (31.25, 38.2)	*−*4.957	<**0.001**
GLB (mg/L)	28.35 (19.33, 33.98)	29.4 (24.93, 35.48)	*−*2.349	**0.019**
A/G	1.21 (1.02, 1.44)	1.1 (0.91, 1.36)	*−*3.095	**0.002**

Bold values in the summary indicate significant p values <0.05.

Q1, Q3, Quartile 1, Quartile 3; ESR, erythrocyte sedimentation rate; CRP, C-reactive protein; IgG, immunoglobulin G; IgA, immunoglobulin A; IgM, immunoglobulin M; WBC, white blood cell; RBC, red blood cell; PLT, platelet; LYM, lymphatic; MO, monocytes; NE, neutrophilic granulocyte; PLR, platelet-lymphocyte ratio; LMR, monocytes- lymphatic ratio; NLR, neutrophil-lymphocyte ratio; SII, systemic immune-inflammation index; BUN, blood urea nitrogen; Cr, creatinine; FBG, fasting blood glucose; ALB, albumin; GLB, globulin; A/G, albumin-globulin ratio.

### 3.3 Peripheral lymphocyte subsets

**Figure 2:**
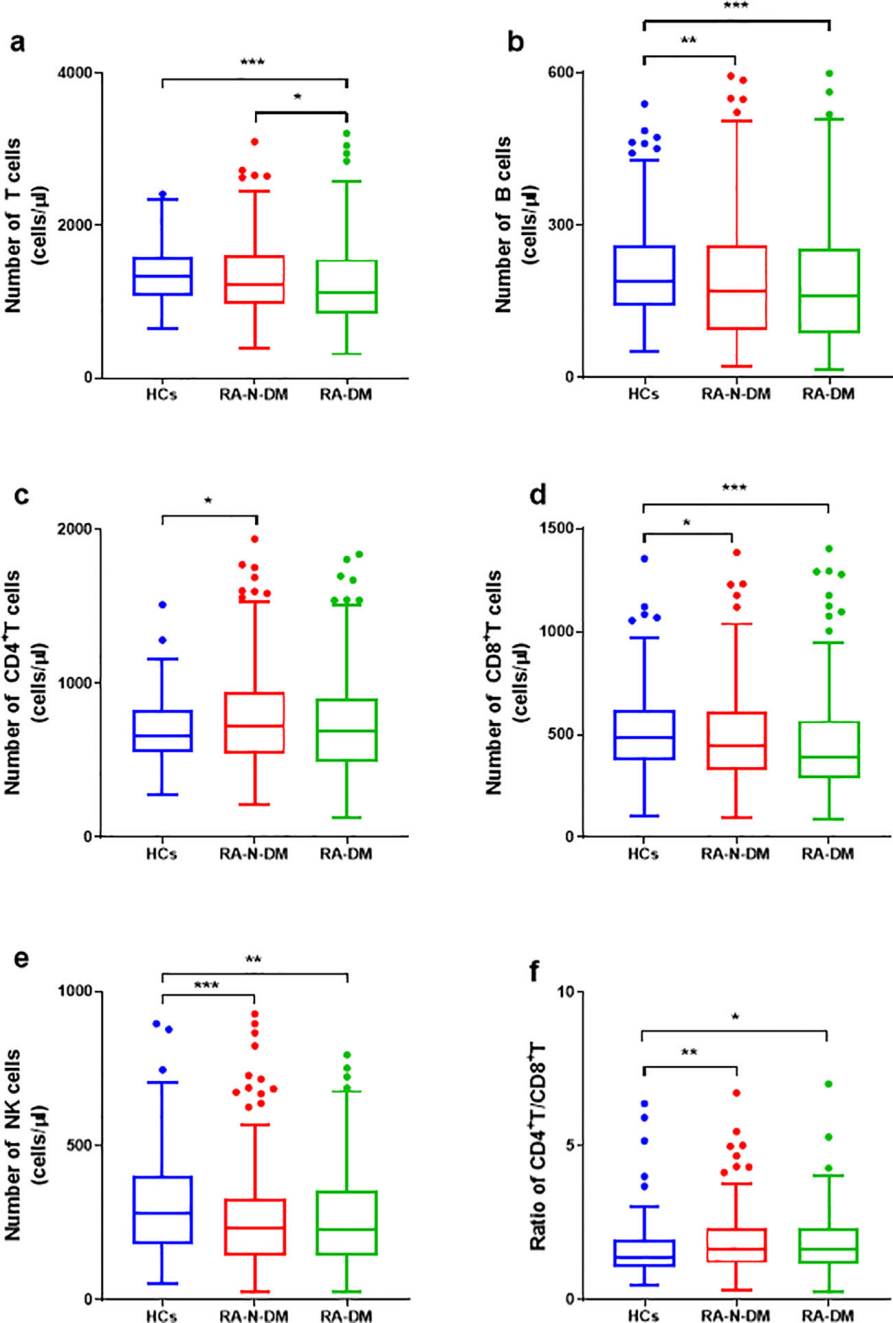
The differences of absolute numbers (cells/µL) of peripheral immune cells among health controls(HCs) and RA patients with and without DM. **(A)** The absolute numbers of T cells in RA-DM (n=160) were significantly lower than HCs (n= 206) and RA-N-DM (n=328). **(B, E)** Absolute numbers of B and NK cells were higher in HCs than both RA-N-DM and RA-DM patients. **(C, D, F)** The levels of CD8^+^T cells were increased while that of CD4^+^T cells reduced in RA patients, resulting in a higher ratio of CD4^+^T / CD8^+^T. Data are presented by median (range), and using the Independent-Samples Kruskal–Wallis H test for statistical analysis. *p < 0.05, **p < 0.01, ***p < 0.001.

Compared with healthy controls, the absolute numbers of B, CD8^+^ T and NK cells in RA-N-DM patients (Z=*−*2.967, *p<*0.01; Z=*−*2.23, *p<*0.05; Z=*−*4.194, *p<*0.001) and RA-DM patients (Z=*−*3.705, *p<*0.001; Z=*−*3.682, *p<*0.001; Z=*−*3.053, *p<*0.01) were significantly reduced, while the proportions of these did not differ significantly (*p*>0.05). This indicates that the proportions of immune cells were not completely parallel to their absolute numbers, and the absolute counts of T cells in RA-DM were significantly lower than those in healthy controls (Z=*−*3.705, *p<*0.001) and RA-N-DM (Z=*−*2.314, *p<*0.05) (**Figure 2**; **Supplementary Figure S1**). These results suggest that rheumatic diseases interfere with the balance of the immune system, which might be associated with the development of complications.

### 3.4 Peripheral CD4^+^ T subsets

**Figure 3:**
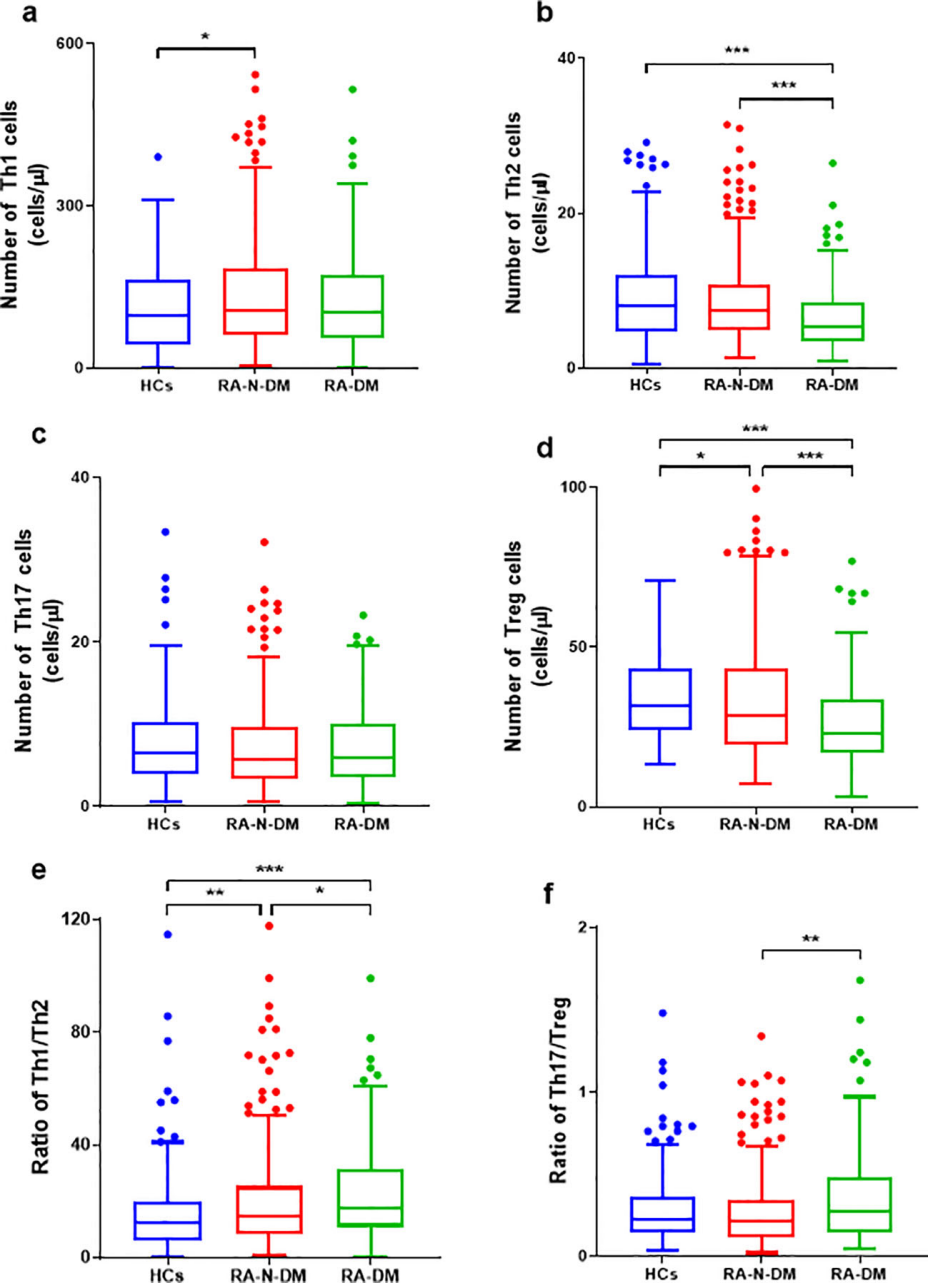
The differences of absolute numbers (cells/μL) of peripheral CD4^+^T subgroups between RA patients (including combined diabetes and not) and healthy controls. **(A, B, D-F)** The absolute numbers of Th2 and Treg cells were significantly reduced in RA-DM patients (n=160), resulting in a higher ratio of Th1/Th2 Th17/Treg. The absolute numbers of Tregs and the ratio of Th1/Th2 in health controls (n=206) were lower than RA patients than, and the Th1 cells was lower than RA-N-DM patients (n=328). **(C)** No statistically significant difference in the Th17 cell numbers between the RA patients and healthy controls. Data are presented by median (range), and using the Independent-Samples Kruskal–Wallis H test for statistical analysis. *p < 0.05, **p < 0.01, ***p < 0.001.

Compared with the healthy controls, the absolute numbers of Th2 cells in the RA-DM group (Z=*−*4.114, *p*<0.001) were significantly reduced, and the proportions of Th2 cells in the RA-N-DM (Z=*−*4.082, *p*<0.001) and RA-DM groups (Z=*−*5.987, *p*<0.001) were significantly decreased, resulting in a higher ratio of Th1/Th2 cells (Z=*−*2.672, *p*<0.01; Z=-4.465, *p*<0.001). Compared to healthy controls, the absolute numbers and proportions of Treg cells in the RA-N-DM (Z=*−*2.431, *p*<0.05; Z=*−*4.113, *p*<0.001) and RA-DM groups (Z=*−*6.041, *p*<0.001; Z=*−*6.395, *p*<0.001) were significantly reduced. Furthermore, compared to the RA-N-DM group, the absolute numbers of Th2 (Z=*−*4.323, <0.001) and Treg cells (Z=*−*3.704, *p*<0.001) and their proportions were significantly reduced in the RA-DM group, which led to an increase in the proportions of Th1/Th2 (Z=*−*2.479, *p*<0.05) and Th17/Treg (Z=*−*3.08, *p <*0.05) cells (**Figure 3**; **Supplementary Figure S2**). This suggests that a reduction in the absolute number and proportion of Th2 and Treg cells affects the activity and development of RA, and is closely related to the occurrence of T2D complications.

### 3.5 Factors associated with combined T2D in patients with RA (Univariable model)

To elucidate the factors associated with T2D in patients with RA, we first performed univariate logistic regression analyses with 21 variables chosen from the results of the previous independent-sample Kruskal-Wallis H test (**Table 3**). In the univariable regression analysis, the risk factors of combined hypertension (OR:1.833, 95% CI: 1.243–2.704, *p<*0.01), history of nephropathy (OR:2.191, 95% CI: 1.125–4.267, *p<*0.05), family history of diabetes (OR:3.354, 95% CI: 1.957–5.747, *p<*0.001), CRP (OR:1.008, 95% CI: 1.003–1.013, *p<*0.01), NLR (OR:1.119, 95% CI: 1.034–1.211, *p<*0.05), GLB (OR:1.023, 95% CI: 1.007–1.038, *p<*0.05) and Th17/Treg ratio (OR:3.419, 95% CI: 1.549–7.547, *p<*0.05) increased the chances of combined T2D in patients with RA. Oppositely, protective factors such as IgG (OR:0.956, 95% CI: 0.915–0.999, *p<*0.05), IgM (OR:0.739, 95% CI: 0.549–0.995, *p<*0.05), ALB (OR:0.9, 95% CI: 0.863–0.939, *p<*0.001), A/G ratio (OR:0.433, 95% CI: 0.236–0.794, *p<*0.01), the levels of Th2 (OR:0.913, 95% CI: 0.873–0.956, *p<*0.001) and Treg cells (OR:0.974, 95% CI: 0.962–0.987, *p<*0.001) reduced the probability of combined T2D in patients with RA.

**Table 3:** A summary of associated factors of RA patients with combined diabetes (Univariable model).

	OR	95% CI	*p*-value
Gender (female/male)	0.98	0.653–1.465	0.914
Age (years)	1.02	0.999–1.041	0.062
Hypertension, n(%)	1.83	1.243–2.704	**0.002**
History of Nephropathy, n(%)	2.19	1.125–4.267	**0.021**
Family history of Diabetes, n(%)	3.35	1.957–5.747	<**0.001**
CRP (mg/L)	1.01	1.003–1.013	**0.001**
IgG (g/L)	0.96	0.915–0.999	**0.043**
IgM (g/L)	0.74	0.549–0.995	**0.046**
PLR	1.00	0.999–1.002	0.74
NLR	1.12	1.034–1.211	**0.005**
ALB (mg/L)	0.90	0.863–0.939	<**0.001**
GLB (mg/L)	1.02	1.007–1.038	**0.003**
A/G	0.43	0.236–0.794	**0.007**
T (cells/µL)	1.00	0.999–1.000	0.133
CD4^+^ T (cells/µL)	1.00	0.999–1.000	0.226
Th2 (cells/µL)	0.91	0.873–0.956	<**0.001**
Th2%	0.56	0.381–0.831	**0.004**
Treg (cells/µL)	0.97	0.962–0.987	<**0.001**
Treg %	0.86	0.771–0.953	**0.004**
Th1/Th2	1.01	1.000–1.022	**0.05**
Th17/Treg	3.42	1.549–7.547	**0.002**

Bold values in the univariable model indicate significant p values <0.05.

CRP, C-reactive protein; PLR, platelet-lymphocyte ratio; NLR, neutrophil-lymphocyte ratio; ALB, albumin; GLB, globulin; A/G, albumin-globulin ratio; T, T lymphocyte; Th1, T helper 1 cells; Th2, T helper 2 cells; Treg, Regulatory cells.

### 3.6 Factors associated with combined T2D in patients with RA (multivariable model)

**Figure 4:**
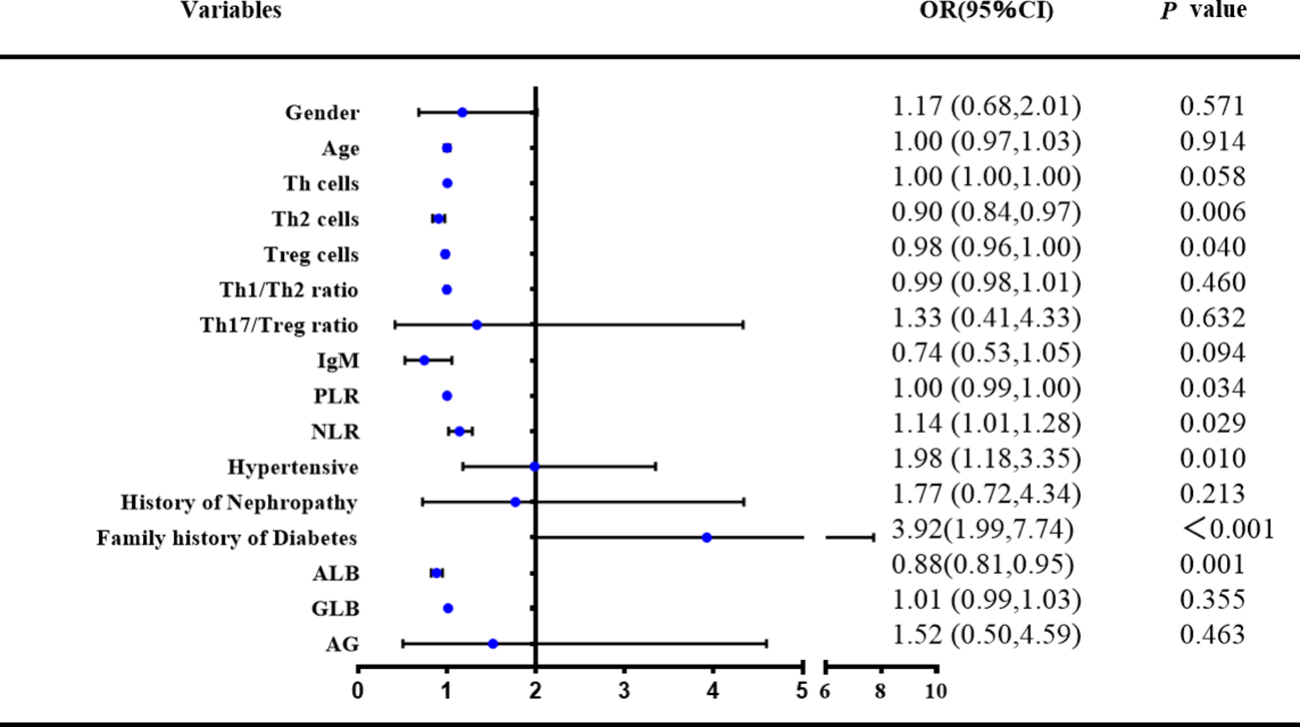
Associated factors of combined diabetes in RA patients (Multivariable model).

Based on the results of the univariate logistic regression analysis and, on this basis, through the analysis of previous research results and the summary of long-term clinical experience, after adjusting for confounding factors, a multivariate logistic regression analysis was conducted on 16 variables (**Figure 4**). The risk factors for RA with diabetes included decreased PLR levels (OR:0.997, 95% CI: 0.994–1, p*<*0.05), ALB (OR:0.881, 95% CI: 0.819–0.946, p*<*0.01), increased NLR levels (OR:1.139, 95% CI: 1.014–1.281, p*<*0.05), fewer Th2 (OR:0.902, 95% CI: 0.837–0.971, p*<*0.01) and Treg cells (OR:0.978, 95% CI: 0.957–0.999, p*<*0.05), increased combined hypertension (OR:1.984, 95% CI: 1.177–3.345, p*<*0.05), and family history of diabetes (OR:3.923, 95% CI: 1.989–7.736, p*<*0.001).

## 4 Discussion

This study identified that certain risk factors, especially immune dysfunction mediated by abnormal numbers of Th2 and Treg cells, may be conducive to the occurrence and development of T2D in patients with RA. A continuous longitudinal population study to explore complications and CD4^+^ T cell subsets in patients with RA may provide new insights into the mechanism of RA complications and provide new ideas for early clinical prevention and treatment.

Among the patients with RA included in this study, the proportion of patients with T2D was approximately 15.6%, and this high prevalence is partly explained by the higher average age and duration of disease in the enrolled patients or the smaller sample size data bias. Studies have shown an increase in average age, BMI, duration of hospitalization, and incidence of hypertension and nephropathy in RA patient with concomitant T2D (14, 15), which is consistent with the findings of this study. Among them, the occurrence of obesity and the increase of BMI may be related to the insulin resistance of type 2 diabetes (16, 17). Notably, poor health, poor exercise capacity, secondary high body weight, and various metabolic organ dysfunctions, which eventually lead to insulin resistance, may be responsible for the increased incidence of T2D and cardiovascular complications in patients with RA (18). T2D has underlying genetic susceptibility (19); as observed in this study, RA patients with T2D also have a higher percentage of a family history of diabetes. Body weight, body mass index (BMI), long disease duration, and family history of diabetes were noteworthy risk factors for RA patients with T2D.

Elevated CRP has not only been observed in patients with RA, but may also be associated with the development of induced insulin resistance and T2D by inhibiting insulin signaling (20, 21). More precisely, CRP induces insulin resistance by activating the MAPK signaling pathways or interfering with the IRS/PI3K/AKT signaling pathway (insulin receptor substrate/phosphatidylinositol 3 kinase/AKT) (22, 23). Consistent with this mechanism, elevated CRP levels have been observed in RA patients with T2D. IgG is deposited in the joints of patients with RA and induces arthritis via its effect on osteoclast production (24). Other studies have shown that a decrease in IgG and other immunoglobulins is associated with insulin resistance (25), which may explain the decrease in IgG and IgM levels observed in RA patients with T2D in this study. In addition, a decrease in immunoglobulin levels may also indicate immune impairment caused by hypoglycemia (26). PLR is an inflammatory index used to predict chronic complications of T2D, and an elevated PLR in patients with type 2 diabetes may reflect the underlying inflammatory burden of the disease (27, 28). In a recent study, remission of the disease and correction of immune disorders in RA patients reduced the incidence of HBP, T2D, and other comorbidities such as MetS and cardiovascular benefits (29). In addition, the underlying chronic low-grade inflammatory state is exacerbated by worsening glycated hemoglobin (HbA1c) levels as a result of poor diabetes control; therefore, inflammatory markers, including PLR, are increased. In the logistic regression analysis adjusted for covariates in this study, PLR was a risk factor for T2D in patients with RA. In this study, ALB, a risk factor for RA patients with T2D, and A/G were significantly lower and GLB was significantly higher in RA patients with T2D, suggesting that T2D plays a crucial role in maintaining glucose homeostasis, insulin clearance, and inflammatory cytokines (29). RA also causes progressive liver damage, which may be associated with vasculitis and hepatocyte amyloidosis caused by RA (30).

Multiple lymphocyte subsets, such as T and B cells are heavily involved in the development of joint dysfunction and systemic inflammation in RA (31). In patients with early RA, the number of T cells, CD80^+^, memory B cells, and NK cells was significantly reduced compared to that in HC, and there was also a trend towards a decrease in the number of CD8^+^ T cells (32, 33). Consistent results were also observed in our study; however, there was no statistical difference in the proportions of B and CD8^+^ T cells between patients compared to HCs, suggesting that the absolute numbers and proportions may not be exactly parallel. Moreover, abnormal cellular immune function plays an indispensable role in the pathogenesis of T2D. T cells and their subsets, which play the most important roles in cellular immunity, are closely related to changes in blood sugar and immune functions (34, 35). Brooks-Worrell et al. demonstrated that T cells are involved in autoimmunity-mediated insulin resistance in a cross-sectional analysis of 322 patients with T2D (36). In this study, compared to RA patients without T2D, patients with T2D had a decreased absolute number of T cells and an increased proportion of CD4^+^ T cells, suggesting that T cells, especially CD4^+^ T subsets, are involved in the occurrence and development of T2D in patients with RA.

Naive CD4^+^ T cells can differentiate into different cell types under the action of antigen-presenting cells (APC). An imbalance in the function and number of these cells leads to abnormalities in the cellular and humoral immunity (37, 38). Previous studies have suggested that the number of Th1 and Th17 cells increases, and the number of Th2 and Treg cells decreases in patients with RA during the active stage of the disease, resulting in an increase in the ratio of Th1/Th2 and Th17/Treg (39–41), which is consistent with our observations. Humoral immune disorders often lead to the overactivation of autoantigen T and B cells, resulting in the deposition of immune complexes in synovial tissue, persistent synovitis, and joint destruction (42).

In this study, the absolute numbers and proportions of Th2 and Treg cells were significantly lower in patients with T2D than in patients without T2D. Previous studies have shown that Th2 cells produce anti-inflammatory factors, such as IL-4, IL-5, IL-6, IL-9, IL-10, and IL-13 to alleviate the inflammatory response, whereas Treg cells inhibit the inflammatory response by inhibiting Th1 and Th17 cells (43). In fact, IL-1, as a common inflammatory pathway of RA, atherosclerotic heart disease, T2D and other systemic diseases, plays a special role in the pathological mechanism of RA combined with T2D (44). Studies have shown that IL-1 inhibition is beneficial for both RA patients and T2D patients, and may become a new targeted treatment option (45). Winer et al (46). detected the number and proportion of Th1, Th17, and Treg cells in the adipose tissue of obese mouse models induced by a high-fat diet, and found that the number of Th1 and Th17 cells in obese mice increased, whereas the number of Treg cells decreased, confirming that Th1 and Th17 cells induce insulin resistance, which can be reversed by Treg cells. Further examination of the CD4^+^ T cell subset levels in the peripheral blood and adipose tissue of newly diagnosed T2D patients showed that CD4^+^ T cells tended to be polarized into pro-inflammatory Th1 and Th17 cells in T2D patients, ultimately leading to inflammation and insulin resistance, underscoring the crucial role of CD4^+^ T subpopulations in the pathogenesis of T2D (46). The results of this study suggest that secondary T2D in patients with RA may be related to reduced anti-insulin resistance caused by decreased Th2 and Treg cells, which was also confirmed in the logistic regression analysis with the multivariable model. This may provide a new possibility for predicting the risk factors for RA in patients with T2D and explain its pathological features.

This study was based on single-center and retrospective methods, with a small sample size and limited comprehensiveness in describing the risk factors for T2D in patients with RA. This should be further developed into a multicenter prospective study to facilitate a more objective and comprehensive research. In this study, patients taking hormones and DMARDs for six months were excluded from the enrollment phase to reduce the effect on peripheral blood lymphocyte levels. However, because these drugs are widely used in clinical treatments, further studies should be conducted to determine their effects on secondary T2D in patients with RA.

## 5 Conclusion

In patients with RA, BMI, cardiovascular comorbidities, systemic inflammatory status, liver function, and lymphocyte levels (particularly Th2 and Treg levels) influence the risk of developing T2D. Testing circulating lymphocyte populations should be a part of the routine management of RA.

## Data Availability

The original contributions presented in the study are included in the article/**Supplementary Material**. Further inquiries can be directed to the corresponding author.
