# Supplementary material for: Risk factors for type 2 diabetes mellitus in Chinese rheumatoid arthritis patients from 2018 to 2022: a real-world, single-center, retrospective study
Source: Front Immunol. 2024 Oct 4;15:1445639. doi: 10.3389/fimmu.2024.1445639 (PMC11486693; doi:10.3389/fimmu.2024.1445639)
Supplement: Supplementary file 1 [file DataSheet1.docx]

Supplementary Material

# Supplementary Figures

**Supplementary Figure 1.** Comparison of the proportions of peripheral immune cells between RA-N-DM (n = 328) and RA-DM (n=160) patients and healthy controls (n = 106). (b, d) The proportions of B and CD8^＋^ T cells were not significantly different among the RA-N-DM and RA-DM and healthy controls. (a, c, e) The proportions of NK cells in RA-N-DM patients were lower and the proportions of T cells and CD4^＋^ T cells were higher than healthy controls. The proportions of CD4^＋^ T cells in RA-DM patients were significantly higher than those in healthy controls. While there was no statistical difference between RA-N-DM and RA-DM patients in terms of the proportions of T and NK cells. Data were calculated as median (range) and compared using the Mann–Whitney U test.

**Supplementary Figure 2.** Comparison of the proportions of peripheral CD4^+^ T subgroups between RA-N-DM (n = 328) and RA-DM (n=160) patients and healthy controls (n = 106). (b, c, d) The proportions of Th2 and Th17 and Treg cells in RA-N-DM patients were significantly higher than RA-DM patients and lower than health controls. The proportions of Th2 and Treg cells were significantly lower in RA-DM patients than in health controls. (a) The proportions of Th1 cells were not significantly different among the RA patients and healthy controls. Data are presented as median (range) and were compared using the Mann–Whitney U test.

**2. Supplementary Figures**

Supplementary Table 1. The results of power analysis

| **Variables** | **RA-N-DM(N=328)** | **RA-DM(N=160)** | **Power** |
| --- | --- | --- | --- |
| **Hypertension, n(%)** | 103（31.4） | 73（45.6） | 0.862 |
| **History of Nephropathy(%)** | 19(5.8) | 19(11.9) | 0.641 |
| **Family history of Diabetes, n(%)** | 27(8.2) | 37(23.1) | 0.99 |
| **Disease duration(years), mean(SD)** | 7.67±7.67 | 11.64±10.32 | 0.99 |
| **Weight(kg), mean(SD** | 60.28±9.82 | 65.07±8.4 | 1 |
| **BMI mean(SD)** | 21.76±5.41 | 24.52±6.74 | 0.994 |
| **NLR** | 2.99±1.92 | 3.87±4.15 | 1 |
| **CRP(mg/L)** | 29.73±33.48 | 45.22±59.97 | 0.857 |
| **IgG** | 13.66±4.92 | 12.57±5.52 | 1 |
| **IgM** | 1.45±0.92 | 1.27±0.76 | 1 |
| **ALB** | 29.73±33.48 | 45.22±59.97 | 1 |
| **GLB** | 13.66±4.92 | 12.57±5.52 | 1 |
| **A/G** | 1.45±0.92 | 1.27±0.76 | 1 |
| **Th2#** | 8.88±6.14 | 6.61±4.27 | 99.7 |
| **Th2** | 1.14±0.59 | 0.97±0.53 | 1 |
| **Treg#** | 33.71±20.55 | 26.02±14.18 | 99.8 |
| **Treg** | 4.43±2.13 | 3.84±1.79 | 1 |
| **Th1/Th2** | 19.67±17.59 | 23.16±17.85 | 1 |
| **Th17/Treg** | 0.27±0.22 | 0.4±0.72 | 1 |
